# Evolution of EEG Motor Rhythms after Spinal Cord Injury: A Longitudinal Study

**DOI:** 10.1371/journal.pone.0131759

**Published:** 2015-07-15

**Authors:** Eduardo López-Larraz, Luis Montesano, Ángel Gil-Agudo, Javier Minguez, Antonio Oliviero

**Affiliations:** 1 Dpto. Informática e Ingeniería de Sistemas, University of Zaragoza, Zaragoza, Spain; 2 Instituto de Investigación en Ingeniería de Aragón (I3A), Zaragoza, Spain; 3 Biomechanics and Technical Aids Unit, Hospital Nacional de Parapléjicos, Toledo, Spain; 4 Bit&Brain Technologies SL, Zaragoza, Spain; 5 FENNSI Group, Hospital Nacional de Parapléjicos, Toledo, Spain; Hospital Nacional de Parapléjicos—SESCAM, SPAIN

## Abstract

Spinal cord injury (SCI) does not only produce a lack of sensory and motor function caudal to the level of injury, but it also leads to a progressive brain reorganization. Chronic SCI patients attempting to move their affected limbs present a significant reduction of brain activation in the motor cortex, which has been linked to the deafferentation. The aim of this work is to study the evolution of the motor-related brain activity during the first months after SCI. Eighteen subacute SCI patients were recruited to participate in bi-weekly experimental sessions during at least two months. Their EEG was recorded to analyze the temporal evolution of the event-related desynchronization (ERD) over the motor cortex, both during motor attempt and motor imagery of their paralyzed hands. The results show that the α and β ERD evolution after SCI is negatively correlated with the clinical progression of the patients during the first months after the injury. This work provides the first longitudinal study of the event-related desynchronization during the subacute phase of spinal cord injury. Furthermore, our findings reveal a strong association between the ERD changes and the clinical evolution of the patients. These results help to better understand the brain transformation after SCI, which is important to characterize the neuroplasticity mechanisms involved after this lesion and may lead to new strategies for rehabilitation and motor restoration of these patients.

## Introduction

Spinal cord injury (SCI) is a devastating disease with a global incidence of 29.5 per million inhabitants per year, and a prevalence of 485 per million inhabitants [1]. Its main consequence is a loss of motor and sensory function caudal to the level of injury. SCI also results in a progressive brain reorganization, which leads to null or significantly reduced brain activations over the motor cortex during the attempt or imagery of the affected limbs [2–5]. This decrease of activity has been explained by a significant reduction in gray matter observed after a long term SCI [6, 7]. Moreover, motor attempt or imagery of paralyzed movements in SCI patients show the activation of brain areas that normally are not necessary for such tasks [3, 8–10].

Brain reorganization after spinal cord injury consists of a progressive change as a consequence of the deafferentation [11]–unlike other neurological diseases such as stroke or traumatic brain injury where the alterations are directly caused by the insult [12, 13]. Hence, the study of brain evolution after SCI can help to better understand the cortical reorganization experienced by these patients [14]. This kind of longitudinal studies has been broadly used with stroke sufferers, where functional recovery has been linked to neuroplasticity and brain reorganization processes using fMRI [15, 16], MEG [17], or EEG [18]. However, most research about brain plasticity after SCI has been done with cross sectional studies, while longitudinal studies about SCI-related brain changes are not frequent [19]. Namely, two pilot studies longitudinally investigated using fMRI how tetraplegic patients that improved their clinical condition increased their motor cortex (M1) activations during the first year post injury [14], and how paraplegics with no clinical improvements suffered from a significant decrease in M1 activation over time [20]. In this line, a recent longitudinal study showed that the anatomic brain changes suffered by a heterogeneous population of SCI patients (including complete and incomplete, tetra- and paraplegic lesions) were correlated with the patients’ functional recovery [21]. The evaluation and quantification of the SCI brain-induced changes can be useful for different reasons. First, it can have a prognostic value and allow predicting the clinical outcome of SCI patients. Secondly, it could be possible to use it in order to follow-up rehabilitation treatments and to design new rehabilitation strategies. It should also be important to consider that plastic changes of the motor and sensory pathways are not only related to the functional outcome but also with the appearance of other SCI complications, like, for example, pain [22]. Event-related desynchronization (ERD) of motor rhythms is considered a marker of task-induced cortical activity during the execution, imagery, and attempt of movements [4, 23]. Indeed, stronger ERD has been associated with a stronger activation of the motor cortex [24]. Furthermore, the ERD can be easily measured in a non-invasive manner in SCI patients.

Our main hypothesis is that the evolution of the ERD can be used as a marker for cortical reorganization after SCI. To test our hypothesis we longitudinally evaluated the ERD changes over time after complete spinal cord injury. We longitudinally recorded the EEG activity of subacute SCI patients while they performed motor attempt and imagery of their paralyzed hands. We measured the trends of ERD activations over time, and evaluated the relationship between these trends and factors related to the clinical evolution of the patients.

## Methods

### Subjects

Eighteen patients with a subacute cervical spinal cord injury participated in this study (two females, age range 18–67 years, mean±SD 36.3±14.4). Clinical details of each patient are reported in Table 1. Inclusion criteria included (*i*) tetraplegia with no grasping capability, (*ii*) complete lesion between levels C3–C6, and (*iii*) time since injury inferior to one year. Exclusion criteria included (*i*) brain injury, (*ii*) inability to understand the protocol or to provide informed consent, and (*iii*) incomplete lesions between levels C3–C6 and preserved grasping capability. Selected participants met all inclusion and no exclusion criteria. All of them were hospitalized at the *Hospital Nacional de Parapléjicos*, in Toledo (Spain), where the experimentation sessions took place between years 2012 and 2014. They underwent conventional rehabilitation during their participation in the study Patients were duly informed about the study and all of them gave written informed consent before the first session. The experimental procedure was approved by the Ethics Review Board of the Hospital (Comité Ético de Investigación Clínica, Complejo Hospitalario de Toledo, dictum 89/22-07-2011).

**Table 1:** Patients’ information.

								Time since injury (days)							
ID	Age	Year of Birth	Level	ASIA	Etiology	Sex	# Sessions	S1	S2	S3	S4	S5	S6	S7	S8
P01	32	1978	C6	B	Traumatic	Male	7	150	164	178	192	220	235	248	-
P02	34	1977	C4/C5	A	Traumatic	Male	8	156	170	184	198	226	241	254	268
P03	32	1979	C4/C5	A	Traumatic	Male	3	136	150	178	-	-	-	-	-
P04	24	1987	C6	A	Traumatic	Male	6	116	131	144	158	188	207	-	-
P05	55	1956	C3	C	Infectious	Male	1	283	-	-	-	-	-	-	-
P06	53	1958	C3	B	Traumatic	Male	2	223	239	-	-	-	-	-	-
P07	23	1988	C5	B	Traumatic	Male	3	108	127	241	-	-	-	-	-
P08	67	1944	C4/C5	B	Traumatic	Male	2	206	225	-	-	-	-	-	-
P09	25	1987	C4/C5	A	Traumatic	Female	8	76	89	101	117	146	174	181	199
P10	23	1988	C4/C6	A	Traumatic	Male	5	88	101	113	176	193	-	-	-
P11	30	1982	C5/C6	B	Traumatic	Male	2	185	201	-	-	-	-	-	-
P12	45	1967	C4	A	Traumatic	Male	7	159	194	212	236	249	262	275	-
P13	44	1968	C4	A	Traumatic	Male	6	139	163	176	189	202	217	-	-
P14	19	1993	C4	A	Traumatic	Male	6	101	125	138	151	164	179	-	-
P15	56	1957	C5	B	Traumatic	Male	1	120	-	-	-	-	-	-	-
P16	43	1969	C5	A	Traumatic	Male	4	288	302	316	344	-	-	-	-
P17	31	1982	C5	B	Traumatic	Female	6	122	136	150	164	178	215	-	-
P18	18	1995	C6	B	Traumatic	Male	6	118	132	146	160	174	210	-	-

Given is the age and year of birth, the level of injury, ASIA impairment scale (ASIA: American Spinal Injury Association), etiology of lesion, sex and number of sessions recorded. In addition, for each of the patients, the time (in days) since the injury is shown for each of his/her sessions.

### Signal acquisition

EEG was recorded using a commercial gTec system (g.Tec GmbH, Graz, Austria). For patients P01 to P08, 16 active electrodes were recorded, placed at AFz, FC3, FCz, FC4, C5, C3, C1, Cz, C2, C4, C6, CP3, CP1, CPz, CP2 and CP4 (according to the international 10/10 system). For patients P09 to P18, 32 channels were recorded by adding the following 16 positions: FP1, FP2, F7, F3, Fz, F4, F8, T7, T8, P7, P3, Pz, P4, P8, O1 and O2. In both cases, the ground and reference electrodes were placed on FPz and on the left earlobe, respectively. The EEG was digitized at a sampling frequency of 256Hz, high-pass filtered at 0.5 Hz, and power-line notch-filtered to remove the 50Hz line interference.

### Experimental design and Protocol

EEG activity of patients was studied longitudinally, with recordings planned every two weeks approximately. However, availability of the patients conditioned the actual recording dates, and sessions were re-scheduled if they were unable to assist to any planned date. Patients were enrolled in the study as early as possible after their injury according to their clinical availability, in order to increase the probabilities of observing spontaneous recovery in some of them [25]. Patients stayed in the study from their recruitment until they were discharged from the hospital or until they voluntarily decided to stop. Number of sessions recorded for each patient ranged from 1 to 8. Due to the longitudinal nature of the study, we only studied the evolution of brain activity of those patients who participated in the study for at least 2 months with a minimum of 4 sessions to be able to measure the evolution over a relevant period of time (11 of them fulfilled this condition, see Table 1).

The experimental sessions consisted of two different conditions: (*i*) motor attempt (MA) of grasping with the right hand, and (*ii*) motor imagery (MI) of grasping with the right hand. Although both conditions are complex in a status of deafferentation and deeferentation, previous studies have shown that SCI patients are able to distinguish and execute them. The attempt and the imagery of a movement involves some different cortical regions (in addition to some common ones) even in a status of chronic deafferentation [10]. Hence, studying the evolution of the brain activations related to both tasks is important to better understand the cortical reorganization in this population. Prior to the first recording session, all patients were carefully instructed to perform both tasks: for MA they were asked to avoid compensatory movements with the arm, and for MI, they were asked to perform kinesthetic imagery to involve the motor cortex [26]. After the instruction, patients were given some minutes to practice, until they verbally confirmed that they were able to differentiate and perform correctly both tasks (MA and MI).

During the experimental sessions, patients were seated in their wheelchairs, with their right arm sustained by the chair armrest and facing a computer screen which displayed visual cues as guidance for the tasks. Each session was divided into six blocks of 4.5 minutes each: three blocks of MA trials, and three blocks of MI trials. Blocks of MA and MI were alternated. Thirty trials were recorded for each block, resulting in a total of 180 trials (90 for each condition). After each block patients could rest as long as they required. Due to restrictions in patients’ schedule or to fatigue, some sessions were stopped before completing the six blocks. The average number of trials recorded on each session for each task was 80±17 (range 30–90).

The trials were composed by three periods, each one denoted by a visual cue. The first cue indicated the patients to stay relaxed in rest position. The second cue denoted the period for attempt or the imagination of movement. The third cue indicated the rest period. Each cue lasted three seconds, so trial duration was 9 seconds. During the first and second cues the patients were instructed to avoid producing artifacts such as blinks or head movements.

### Clinical assessments

A neurological examination of all the patients was performed according to the ASIA standards [27]. The ASIA motor sub-score (MS) of their right arm was obtained as the sum of the muscle strength of C5 to T1 segments. The strength of each muscle was evaluated by a clinician in a 0–5 scale, making motor score range from 0 to 25 points [27]. Additionally, the functional independence of each patient was measured with the SCIM scale (range 0–100) [28].

MS and SCIM tests were applied on the recruitment of the patients (pre assessment) and when they left the study (post assessment). Their clinical evolution was measured as the difference between the post and pre assessments in both scales.

### Signal preprocessing

EEG signals were segmented into 6-second trials, from -3 to 3 seconds with respect to the presentation of the second cue (initiation of the MA/MI). Trials were bandpass filtered between 1 and 50 Hz using a zero-phase fourth-order Butterworth filter. Artifact rejection was performed by visual inspection of the trials. On average for each subject and session, 66±18 trials (range 22–90) remained after the rejection procedure.

Spatial filtering was performed independently in three areas of interest of the motor cortex, namely in the left hemisphere (LH: computed with channels FC3, C5, C3, C1, CP3 and CP1), midline (ML: channels FCz, Cz and CPz), and right hemisphere (RH: channels FC4, C2, C4, C6, CP2 and CP4). Optimal spatial filters (OSF) were used to obtain the linear combination of the channels of the corresponding areas of interest that maximized the signal to noise ratio (SNR) of the ERD, and removed the reference [29, 30]. Since a different OSF was computed for each session and area, the resulting ERD was invariant to variability across sessions of the measurements at sensor level.

### Event-related desynchronization analysis

We computed the event-related desynchronization (ERD) in *α* and *β* frequency bands to measure the brain activation during the movement attempt and imagery. Notice that the ERD measures the relative power decrease with respect to a baseline period. Hence, ERD is reported with negative values, representing cortical activations. On the other hand, the event-related synchronization (ERS) is a power increase, which is reported with positive values, and is associated to cortical idling [23]. The ERD analysis was computed separately for motor attempt and imagery in the signals obtained with the OSF as descriptors of the brain activity of the LH, ML and RH. The time-frequency representations of each subject and session were obtained using Morlet Wavelets in the frequency range [1–50] Hz [31]. Statistical significance (*α* = 0.05) of the computed ERD was calculated with respect to baseline [-3, 0] using a bootstrap resampling method [29].

### Trend analysis

**Fig 1:**
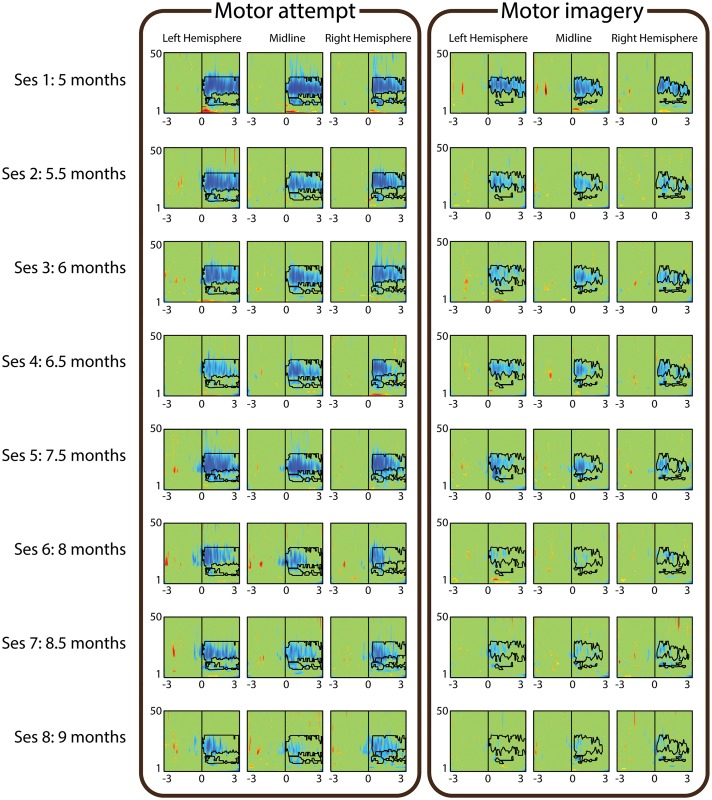
ERD Evolution of patient P02. ERD maps corresponding to motor attempt (left panel) and motor imagery (right panel). The different rows correspond to the 8 sessions, which were registered between 5 and 9 months after the lesion. The three columns of each panel correspond to the ERD of the left hemisphere, midline and right hemisphere. The *x*-axes of each map range from -3 to 3 seconds with respect to the attempt/imagery cue presentation, while the *y*-axes range from 1 to 50 Hz. The *α* and *β* regions of interest obtained on the first session for each channel are drawn on each ERD map.

The evolution of the motor-related activity of each brain area was characterized with a trend analysis in *α* and *β* ERD with respect to the first recorded session of each patient. For each patient and session, the average ERD was computed within two individualized regions of interest: one for *α* (7–13 Hz) and one for *β* (14–30 Hz) frequencies. This analysis was performed for MA and MI, and on each OSF signal separately. The personalized regions of interest were computed from the ERD maps corresponding to the first session as the time-frequency pairs which presented statistically significant activity in the *α* and *β* ranges within time interval [0–3] s. These regions of interest obtained for the first session remained fixed for the computation of the average *α* and *β* ERD of the subsequent sessions (see Fig 1).

Trends of *α* and *β* ERD were calculated as the slope of a regression line fitted to the average ERD values of each session. Significance of the linear trends was measured as the Spearman’s rank correlation coefficient between the ERD of each session and the time since the injury. The obtained p-values were FDR corrected for multiple comparisons. The evolution of each patient was measured individually, and hence, each of them set his/her baseline on the first session.

### Clinico-physiological correlations

We studied the correlation between the ERD trends and factors such as the clinical variables (SCIM and MS) and the initial ERD. In order to evaluate differences in trends related to the clinical evolution of the patients, we defined a new variable to group the subjects according to the presence or absence of relevant clinical improvements. The relevant clinical improvement was defined by a clinical specialist as an increase in more than 10 points in SCIM or more than 5 points in MS. We averaged the trends of the group of subjects with clinical improvement, and of the group without clinical improvement. Subjects with unavailable data in any of the scales were excluded of this analysis.

Moreover, we studied the influence of the initial ERD in its posterior trend. We performed a correlation analysis between the ERD trends and the initial ERD of each subject. Subsequently, the subjects were divided into two groups, depending on the ERD magnitude recorded on their first session. We set a threshold to 20% and considered subjects with a small-ERD if they desynchronized less than that threshold during the first session, or normal-ERD if they desynchronized above that value.

## Results

Table 2 shows the average ERD values over the individualized regions of interest on the left hemisphere for the first session of each subject, when performing MA and MI. These measures were considered as the initial state to subsequently monitor evolution of ERD over time. Notice that, as values of MA and MI in Table 2 correspond to the average ERD within the individualized regions of interest, these values are not comparable.

**Table 2:** Initial ERD of each subject.

	**Motor attempt**		**Motor imagery**	
	**LH *α*-ERD**	**LH *β*-ERD**	**LH *α*-ERD**	**LH *β*-ERD**
P01	-30.1%	-19.6%	-34.7%	-16.9%
P02	-24.5%	-49.3%	-26.1%	-38.4%
P03	3.6%	-4.9%	-20.2%	-15.8%
P04	-26.6%	-26.0%	-6.7%	-24.4%
P05	-42.8%	-40.5%	n.a.	n.a.
P06	-30.2%	-10.7%	-31.0%	-41.6%
P07	-38.0%	-28.9%	-33.7%	-10.5%
P08	-25.1%	-21.9%	-32.6%	-22.9%
P09	-19.9%	-32.6%	-24.1%	-1.8%
P10	-38.0%	-39.3%	-37.2%	-33.0%
P11	-33.7%	8.2%	-39.0%	-16.7%
P12	-38.0%	-49.9%	-32.5%	-38.4%
P13	-9.0%	14.0%	-33.4%	-24.6%
P14	-21.8%	-17.3%	-36.4%	-16.0%
P15	-43.7%	-29.5%	-33.2%	-23.1%
P16	-33.2%	-29.2%	-15.0%	5.8%
P17	-38.7%	-44.1%	-32.9%	-33.9%
P18	-16.9%	-7.5%	-16.2%	-12.8%
**Avg**	**-28.1%**	**-23.8%**	**-28.5%**	**-21.5%**

Initial ERD percentage of the 18 patients on the left hemisphere for motor attempt and imagery. The percentages are computed as the average ERD within the defined *α* and *β* regions of interest. n.a.: not available.

**Fig 2:**
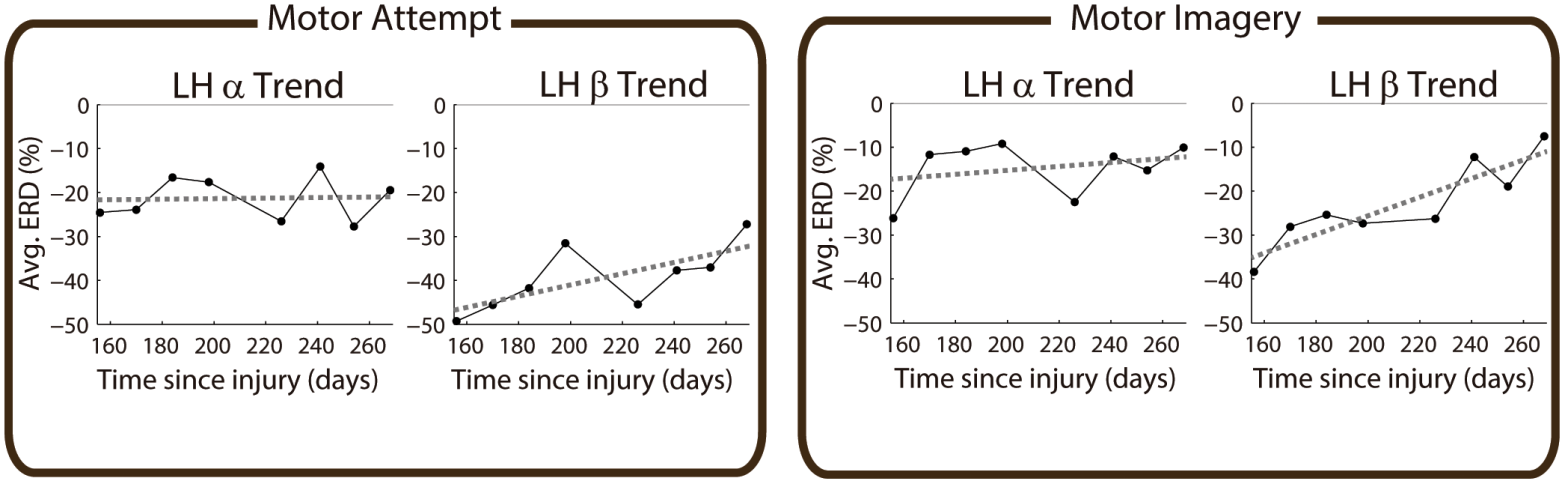
Linear *α* and *β* ERD trends obtained for participant P02 in the left hemisphere. Left and right panels correspond to motor attempt and imagery, respectively. Left and right within each panel correspond to *α* and *β* trends. The black dots of each subplot represent the average ERD obtained in the eight sessions recorded with this patient. The *x*-axes represent the time in days since injury, while the *y*-axes correspond to ERD magnitude.

Fig 1 shows an example of evolution of motor rhythms over four months in a SCI patient with no clinical improvement (P02). Significant ERD for MA and MI decreased from the first recorded session (5 months after the lesion) to the last one (9 months after the lesion) in the three studied brain regions (i.e., LH, ML, RH). The decrease in ERD occurred both in time (as a reduction in ERD duration), and in frequency (as a reduction in the frequency range with significant activity). Fig 2 shows the evolution of average *α* and *β* ERD in the LH for the same participant. For this participant there is a reduction in *β* ERD for MA and MI, shown by the high positive-slope linear trends. On the other hand, *α* ERD has a flat trend for MA, and a small positive trend for MI.

Subjects with less than two months of evolution studied (*n* = 7) were discarded for the analysis of ERD trends. For the remaining 11 subjects, Table 3 presents the slopes of the regression lines fitted for *α* and *β* ERD values in LH, ML and RH for both motor attempt and imagery. Positive slopes represent the tendency of ERD to decrease over time (as the ones of P02 seen in Fig 2), while negative slopes imply an increase in ERD. Trends with statistical significance are underlined, denoting in bold face the significant decreases in ERD, and in italics the significant increase in ERD. Changes in clinical scales are shown in the two central columns as the difference in SCIM and MS scales between the post and the pre evaluation. For the cases in which it was not possible to record the pre or post clinical evaluation, the difference in scales could not be computed, and are represented in the table as n.a. (not available). As can be seen from Table 3, for MA, 25 of the 66 combinations studied showed a significant decrease in ERD, while just 2 showed a significant increase. On the other hand, for MI, 14 showed a significant decrease and 2 showed a significant increase.

**Table 3:** ERD Trends and clinical evolution.

	Motor Attempt								Motor Imagery					
	*α*-Evol			*β*-Evol			Clin. Data		*α*-Evol			*β*-Evol		
	LH	ML	RH	LH	ML	RH	diff SCIM	diff MS	LH	ML	RH	LH	ML	RH
P01	0.06	0.01	**0.18**	0.03	-0.07	-0.01	0	0	0.02	0.03	0.11	-0.06	-0.02	-0.04
P02	0.00	0.01	0.04	**0.13**	**0.17**	**0.09**	1	2	0.04	0.04	**0.10**	**0.21**	**0.15**	**0.14**
P04	**0.20**	0.07	**0.24**	**0.26**	0.07	0.14	n.a.	n.a.	0.01	0.17	-0.02	0.15	-0.04	-0.04
P09	0.04	**0.03**	0.11	**0.12**	-0.08	**0.07**	13	2	**0.13**	-0.18	-0.06	-0.05	0.00	0.01
P10	0.15	**0.15**	**0.17**	-0.07	-0.05	0.01	22	0	-0.05	-0.31	*-0.56*	0.05	-0.17	*-0.53*
P12	*-0.19*	0.04	0.01	-0.01	**0.15**	**0.12**	2	7	-0.04	0.09	0.09	**0.18**	**0.20**	**0.16**
P13	0.06	0.16	*-0.22*	-0.19	0.01	-0.06	n.a.	0	**0.34**	0.34	0.18	0.15	0.22	**0.19**
P14	**0.27**	0.44	0.30	**0.34**	0.16	**0.27**	0	0	0.04	**0.37**	**0.37**	0.12	0.12	0.23
P16	**0.27**	**0.43**	0.31	**0.26**	**0.23**	0.33	n.a.	0	-0.21	0.23	-0.05	-0.07	0.12	0.19
P17	0.03	0.11	**0.17**	**0.21**	**0.27**	**0.21**	4	1	-0.01	-0.10	0.08	0.15	**0.21**	**0.20**
P18	-0.13	-0.01	-0.47	0.05	0.03	0.04	26	1	0.10	-0.27	0.00	0.02	-0.32	0.05

ERD trends and clinical evolution of the 11 analyzed patients. Underlined values represent statistical significance, with bold face/italics representing significant ERD decreases/increases. *α*-Evol: Evolution of the ERD in *α* band. *β*-Evol: Evolution of the ERD in *β* band. LH: Left hemisphere. ML: Midline. RH: Right hemisphere. diff SCIM: Difference (post-pre evaluation) in Spinal Cord Independence Measure (SCIM) scale. diff MS: Difference (post-pre evaluation) in the right arm motor score (MS). n.a.: not available.

**Fig 3:**
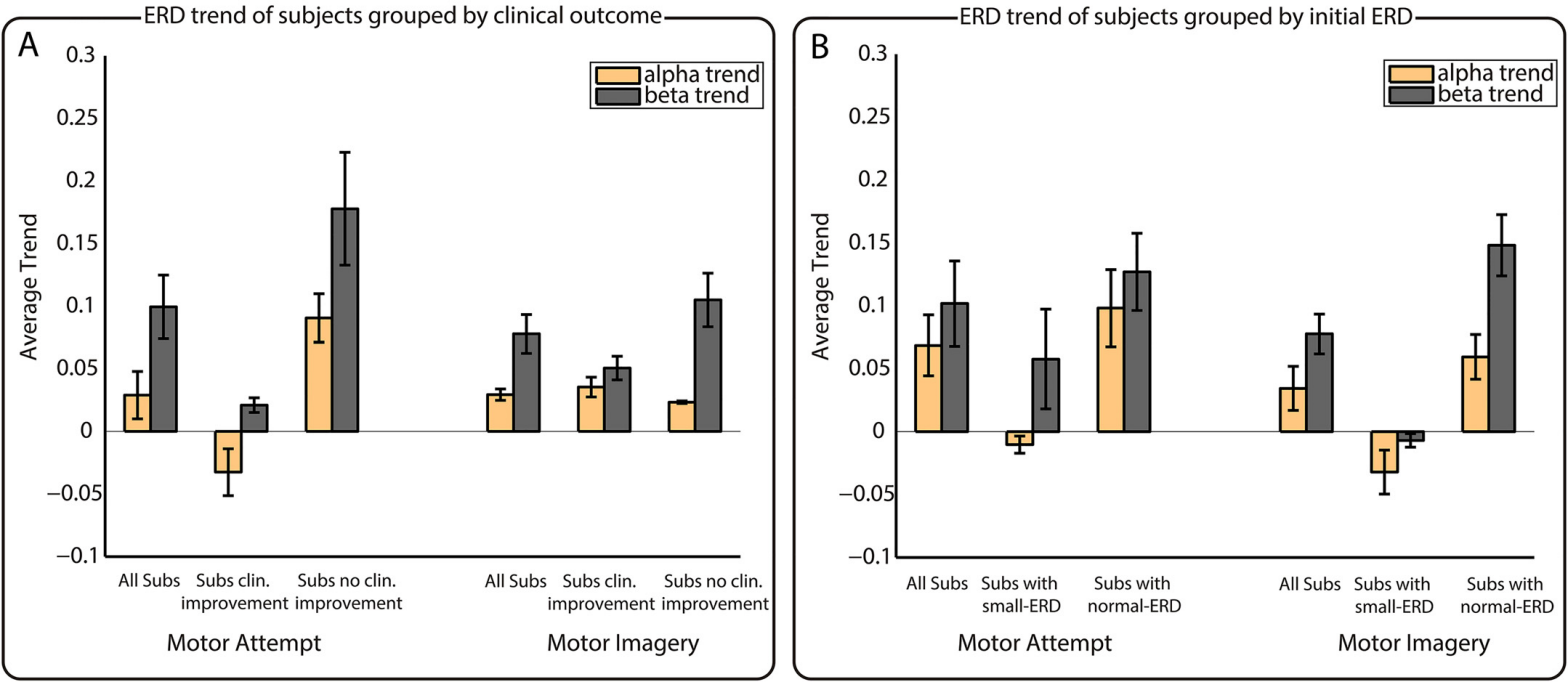
Analyses of grouped ERD trends. Analysis of the ERD trends grouping subjects by clinical outcome (A) and by initial ERD (B). Left/right part of panels A and B correspond to MA/MI, respectively. The *x*-axes of the figures indicate the corresponding groups, while the *y*-axes correspond to the averaged trend of the fitted regression lines.

Top part of Table 4 shows the analysis of correlations between *α* and *β* trends and the clinical variables. We found strong correlations for motor attempt between SCIM change and *β* trend (*r* = −0.47), between MS change and *α* trend (*r* = −0.71), and between the clinical change and *α* (*r* = −0.46) as well as *β* (*r* = −0.63) trends. In all cases the correlations were negative, suggesting that the reduction in ERD over time can be associated to a lack of improvement in clinical condition. On the other hand, for motor imagery there were positive and negative correlations, showing weaker values that were never over 0.4. Fig 3-A shows the trend analysis according to the patients’ clinical evolution. Notice that data of patients P04, P13, and P16 was not considered for this analysis since their clinical information was not complete. The figure shows the averaged ERD trends for the eight subjects for which all the clinical information was available, so as for the groups with (*n* = 4) and without (*n* = 4) relevant clinical improvements for both MA and MI. The figure shows that, on average, *α* trends were lower than *β* trends. For MA, subjects with no clinical improvement presented higher trends, reflecting a faster reduction of ERD over time (left part of Fig 3-A). In contrast, subjects with clinical improvements showed a negative trend in *α* (meaning *α* ERD increase over time), and a small positive trend for *β*. For MI, these results were not so evident, and it is just in the case of *β* trends that can be seen how subjects with clinical improvements showed slightly smaller trends than the ones without improvements (right part of Fig 3-A). However, as the number of subjects available for these analyses was small, statistical comparisons provided no significant results.

**Table 4:** Correlation between ERD trends and clinical variables (top) and initial ERD (bottom).

	Motor Attempt				Motor Imagery			
	*α* Trend		*β* Trend		*α* Trend		*β* Trend	
	r	p-val	r	p-val	r	p-val	r	p-val
SCIM	-0.17	0.69	-0.47	0.24	0.24	0.57	-0.4	0.32
MS	-0.72	0.02	-0.15	0.68	-0.12	0.74	0.4	0.25
Clin. Change	-0.46	0.25	-0.63	0.09	0.1	0.81	-0.28	0.5
Init-ERD	-0.02	0.96	-0.29	0.38	-0.2	0.55	-0.82	0.002

Spearman’s correlations between the ERD trends and the two clinical variables (i.e., SCIM and MS), and between the ERD trends and the initial ERD. *α* Trend: ERD trend in *α* band. *β* Trend: ERD trend in *β* band. r: Spearman’s correlation coefficient. p-val: P-value. SCIM: Spinal Cord Independence Measure. MS: Motor score. Clin. Change: Clinical change. Init-ERD: Initial ERD.

Bottom part of Table 4 shows the analysis of correlations between ERD trends and initial ERD. This comparison showed a significant correlation just between *β* trend and initial *β* ERD for motor imagery. Fig 3-B shows the ERD trends averaged for all subjects, so as for groups with small-ERD and normal-ERD for MA and MI. The number of subjects in each group varied for each comparison (*n*_*small*−*ERD*, *α*, *MA*_ = 3, *n*_*normal*−*ERD*, *α*, *MA*_ = 8; *n*_*small*−*ERD*, *β*, *MA*_ = 4, *n*_*normal*−*ERD*, *β*, *MA*_ = 7; *n*_*small*−*ERD*, *α*, *MI*_ = 3, *n*_*normal*−*ERD*, *α*, *MI*_ = 8; *n*_*small*−*ERD*, *β*, *MI*_ = 5, *n*_*normal*−*ERD*, *β*, *MI*_ = 6). Despite only 1 out of the 4 comparisons performed showed strong correlations (see bottom row in Table 4), this analysis showed that subjects with a small-ERD in their first session also presented smaller trends, specially in *α* for MA and *α* and *β* for MI, where we observed negative trends for the small-ERD group.

**Fig 4:**
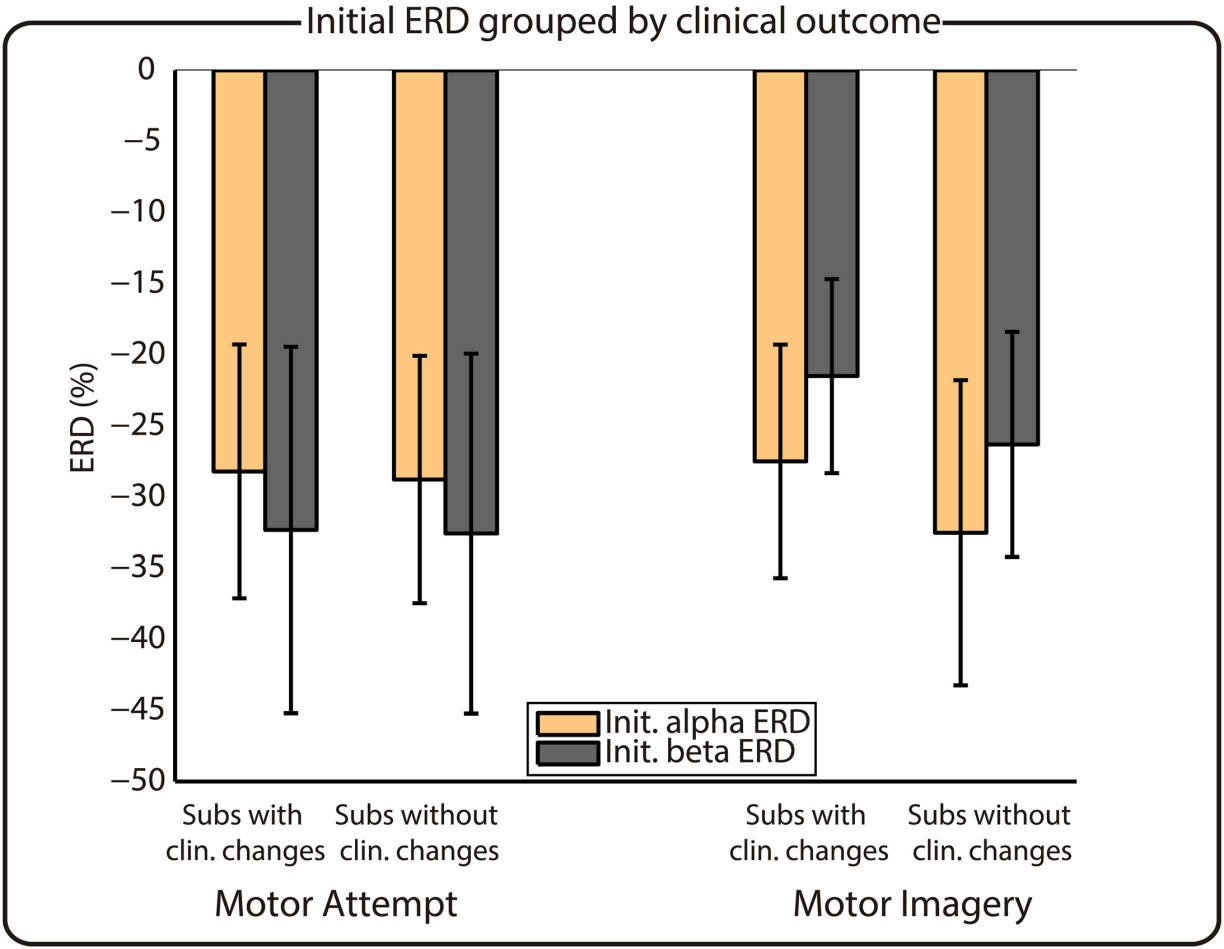
Initial ERD values of subjects grouped by clinical outcome. Left/right part of the figure corresponds to MA/MI, respectively. The *x*-axis indicates the group, while the *y*-axis shows the ERD percentage.

The two previous analyses showed that subjects with clinical improvements and subjects with a small-ERD present smaller trends. Therefore, we explored if there is any relationship between these two factors (i.e., clinical change and initial ERD) by comparing the average initial ERD values of groups of subjects with and without clinical changes. Fig 4 shows that there are no differences in ERD dependent upon the clinical outcome, neither for MA nor for MI.

## Discussion

This paper reported for the first time the temporal evolution of the event-related desynchronization produced by motor attempt and imagery of the upper limb in patients with tetraplegia caused by SCI. Our results show that there is a strong association between the clinical progression of a SCI patient and the trend of his/her ERD activations during the attempt of motion.

These findings are in line with previous literature, which studied neuroplastic changes in subacute SCI patients using different neuroimaging techniques. Jurkiewicz et al. demonstrated using fMRI that paraplegic patients with persisting paralysis suffered from a significant decrease in activations of M1 and associated cortical sensorimotor areas over time [20]. Our results also provided evidence that patients with persisting paralysis suffer from a decrease in cortical activation, reflected as a faster reduction in *α* and *β* ERD during MA. On the other hand, for patients who improved their clinical condition, we observed an increase in cortical activation in *α* frequency and a small decrease in *β* frequency over time during MA. This agrees with the results presented in [14], which showed using fMRI how brain activation of tetraplegic patients in M1 increased over time until reaching similar patterns to healthy controls, as they improved their motor function. This evolution towards brain patterns of healthy subjects with recovery was also shown in motor-related cortical potentials by Green et al., who demonstrated using EEG how the patients’ motor potentials–which were initially located more posteriorly than in healthy controls–were displaced towards anterior regions as they improved their motor function [32].

Interestingly, we observed that the strong correlations that we found between clinical evolution and the ERD trends of MA were not present for the trends of MI, which suggests that the brain networks responsible of attempt and imagery of movements can suffer from different neuroplastic evolutions. This result is in line with previous studies which have shown that significant differences are present in the neural networks responsible of MA and MI after chronic SCI [3, 10, 33].

Our results also showed that subjects with a small initial ERD presented smaller trends. This might be explained by a flooring effect in ERD evolution, suggesting that smaller ERDs are not so prone to be lowered, as they already reflect a small brain activation. In addition, our results also found no relationship between the initial ERD and the clinical evolution of the patients. This latter observation suggests that ERDs cannot be used as a prognostic variable of clinical evolution (at least in the time window we evaluated).

We would like to underline some potential limitations of our study. Most of the patients were under neuroactive medication that can have some influence on brain activity. The effects of these drugs cannot be avoided in such kind of studies as it is ethically unacceptable to modify/withdraw the treatment for the whole period of the study (months). On the other hand, we consider that the longitudinal design limits the impact of the drugs on our results as all the patients maintain most of the drugs unchanged during the whole study period. Regarding the tasks used to induce the motor brain activations (i.e., motor attempt and imagery), there was no way to quantitatively assess the correct performance. However, we carefully instructed the patients to perform both of them. Their positive answers when asked for their ability to perform both tasks correctly, together with the differences we observed in the brain activations, make us consider that the patients were able to perform both tasks properly. Another potential limitation of the study is that we correlated the clinical evolution with the ERD. Clinical improvement is probably related to changes at brain and spinal levels, whereas ERD evaluate more specifically changes that happens at brain level. However, we consider that this limitation does not affect the interest of our results.

Our main results are that ERD is able to quantify the brain neuroplastic changes that parallel the clinical evolution. This means that a measure of how the brain is activated/deactivated in the subacute phase of SCI during a motor task is–at least in part–marker of the clinical evolution. SCI studies in animal models showed that the deafferented cortex suffers alterations of the oscillatory characteristics [11]. Speculatively, we can suggest that deafferentation alters the intrinsic responsiveness of the sensorimotor system. Moreover, it is conceivable that this pathological responsiveness changes over time to drive (or to be driven) by the functional reorganization of the brain induced by a lesion.

Brain reorganization after central nervous system lesions play a role in the recovery and rehabilitation of sensory and motor dysfunction. This reorganization can be adaptive (useful for regaining the lost function) or maladaptive (useless to regain lost functions or even it can worsen the clinical pictures) [22]. As these neuroplastic changes constitute a dynamic process, every technique able to monitor the brain reorganization can be useful in the understanding of what is happening in the brain during the recovery after this lesion. The understanding of the cortical reorganization will allow to personalize rehabilitation strategies, for this reason there is a great need of markers that can be easily and non-invasively obtained from the patients. The ERD can be one of these markers.

In this study we focused on analyzing the brain activations of SCI patients suffering from complete tetraplegia. As brain dynamics can vary according to the level and completeness of lesion [19, 21, 34] further research should be done to evaluate the influence of the typology of the lesion in the neuroplasticity process. Our study also evidenced an important logistic issue for longitudinal studies with this population of highly dependent patients, which is the elevated rate of subjects dropping out before two months of recordings (7/18). Of the remaining 11 patients, complete clinical evaluations was just available for 8 of them (4 who showed clinical improvements and 4 who did not). This impeded obtaining statistically significant results. Hence we consider that future research to completely characterize brain dynamics after SCI should consider an elevated number of patients, and extend the length of the study to the first years after the lesion, to allow measuring the approximate time when the brain activations related to motor commands reach the chronic state.

One major practical consequence of the evolution of motor rhythms in this population is the impact of these changes in the use of EEG-based brain machine interfaces (BMI) for neurorehabilitation or functional compensation. These systems will need to cope with the variations of the rhythms and monitor the effect the interventions may have on them. Although some studies have demonstrated that both MA and MI can be detected using EEG in complete SCI subjects [35, 36], patients with a severe loss of ERD might be unable to use a BMI based on these rhythms. Hence, such patients may require training interventions such as the ones proposed by [37] and [38] to enhance the motor-related brain activations.
